# Supplementary material for: Pyruvate Dehydrogenase Kinase 1 inhibition mediated oxidative phosphorylation enhancement in cartilage promotes osteoarthritis progression
Source: BMC Musculoskelet Disord. 2023 Jul 20;24:597. doi: 10.1186/s12891-023-06585-6 (PMC10357736; doi:10.1186/s12891-023-06585-6)
Supplement: Supplementary file 1 — Additional file 1. [file 12891_2023_6585_MOESM1_ESM.zip › KEGG.Gsea.1653622432464/KEGG_NEUROACTIVE_LIGAND_RECEPTOR_INTERACTION.html]

Details for gene set KEGG\_NEUROACTIVE\_LIGAND\_RECEPTOR\_INTERACTION[GSEA]

|  || Dataset | 55457\_collapsed\_to\_symbols.cls.cls #OA\_versus\_normal.cls.cls #OA\_versus\_normal\_repos |
| Phenotype | cls.cls#OA\_versus\_normal\_repos |
| Upregulated in class | normal |
| GeneSet | KEGG\_NEUROACTIVE\_LIGAND\_RECEPTOR\_INTERACTION |
| Enrichment Score (ES) | -0.27663818 |
| Normalized Enrichment Score (NES) | -1.3644912 |
| Nominal p-value | 0.01010101 |
| FDR q-value | 0.2516273 |
| FWER p-Value | 0.999 |
Table: GSEA Results Summary

  

Fig 1: Enrichment plot: KEGG\_NEUROACTIVE\_LIGAND\_RECEPTOR\_INTERACTION      
 Profile of the Running ES Score & Positions of GeneSet Members on the Rank Ordered List

  

| SYMBOL | TITLE | RANK IN GENE LIST | RANK METRIC SCORE | RUNNING ES | CORE ENRICHMENT || 1 | CHRNB1 | cholinergic receptor nicotinic beta 1 subunit [Source:HGNC Symbol;Acc:HGNC:1961] | 165 | 0.844 | -0.0011 | No |
| 2 | DRD1 | dopamine receptor D1 [Source:HGNC Symbol;Acc:HGNC:3020] | 392 | 0.709 | -0.0090 | No |
| 3 | PTGER4 | prostaglandin E receptor 4 [Source:HGNC Symbol;Acc:HGNC:9596] | 403 | 0.705 | 0.0002 | No |
| 4 | ADORA3 | adenosine A3 receptor [Source:HGNC Symbol;Acc:HGNC:268] | 537 | 0.645 | -0.0012 | No |
| 5 | HTR2B | 5-hydroxytryptamine receptor 2B [Source:HGNC Symbol;Acc:HGNC:5294] | 562 | 0.636 | 0.0059 | No |
| 6 | ADRB2 | adrenoceptor beta 2 [Source:HGNC Symbol;Acc:HGNC:286] | 596 | 0.625 | 0.0121 | No |
| 7 | LPAR6 | lysophosphatidic acid receptor 6 [Source:HGNC Symbol;Acc:HGNC:15520] | 661 | 0.603 | 0.0156 | No |
| 8 | NR3C1 | nuclear receptor subfamily 3 group C member 1 [Source:HGNC Symbol;Acc:HGNC:7978] | 674 | 0.599 | 0.0231 | No |
| 9 | TSPO | translocator protein [Source:HGNC Symbol;Acc:HGNC:1158] | 692 | 0.595 | 0.0302 | No |
| 10 | AVPR1B | arginine vasopressin receptor 1B [Source:HGNC Symbol;Acc:HGNC:896] | 865 | 0.552 | 0.0244 | No |
| 11 | GRIN2A | glutamate ionotropic receptor NMDA type subunit 2A [Source:HGNC Symbol;Acc:HGNC:4585] | 1054 | 0.506 | 0.0167 | No |
| 12 | GLRB | glycine receptor beta [Source:HGNC Symbol;Acc:HGNC:4329] | 1382 | 0.445 | -0.0029 | No |
| 13 | CHRNA4 | cholinergic receptor nicotinic alpha 4 subunit [Source:HGNC Symbol;Acc:HGNC:1958] | 1393 | 0.444 | 0.0026 | No |
| 14 | PTGFR | prostaglandin F receptor [Source:HGNC Symbol;Acc:HGNC:9600] | 1416 | 0.441 | 0.0071 | No |
| 15 | SSTR4 | somatostatin receptor 4 [Source:HGNC Symbol;Acc:HGNC:11333] | 1597 | 0.414 | -0.0013 | No |
| 16 | P2RX4 | purinergic receptor P2X 4 [Source:HGNC Symbol;Acc:HGNC:8535] | 1827 | 0.381 | -0.0140 | No |
| 17 | GABRA6 | gamma-aminobutyric acid type A receptor subunit alpha6 [Source:HGNC Symbol;Acc:HGNC:4080] | 1831 | 0.381 | -0.0089 | No |
| 18 | HTR2A | 5-hydroxytryptamine receptor 2A [Source:HGNC Symbol;Acc:HGNC:5293] | 1847 | 0.379 | -0.0047 | No |
| 19 | CCKAR | cholecystokinin A receptor [Source:HGNC Symbol;Acc:HGNC:1570] | 1849 | 0.379 | 0.0006 | No |
| 20 | PTGDR | prostaglandin D2 receptor [Source:HGNC Symbol;Acc:HGNC:9591] | 1904 | 0.372 | 0.0016 | No |
| 21 | GABRA4 | gamma-aminobutyric acid type A receptor subunit alpha4 [Source:HGNC Symbol;Acc:HGNC:4078] | 1914 | 0.371 | 0.0061 | No |
| 22 | C3AR1 | complement C3a receptor 1 [Source:HGNC Symbol;Acc:HGNC:1319] | 1933 | 0.368 | 0.0099 | No |
| 23 | GABRB1 | gamma-aminobutyric acid type A receptor subunit beta1 [Source:HGNC Symbol;Acc:HGNC:4081] | 2036 | 0.357 | 0.0069 | No |
| 24 | GABRQ | gamma-aminobutyric acid type A receptor subunit theta [Source:HGNC Symbol;Acc:HGNC:14454] | 2042 | 0.357 | 0.0115 | No |
| 25 | PTGIR | prostaglandin I2 receptor [Source:HGNC Symbol;Acc:HGNC:9602] | 2055 | 0.356 | 0.0156 | No |
| 26 | GABRA3 | gamma-aminobutyric acid type A receptor subunit alpha3 [Source:HGNC Symbol;Acc:HGNC:4077] | 2087 | 0.351 | 0.0181 | No |
| 27 | GRIA2 | glutamate ionotropic receptor AMPA type subunit 2 [Source:HGNC Symbol;Acc:HGNC:4572] | 2342 | 0.322 | 0.0026 | No |
| 28 | GZMA | granzyme A [Source:HGNC Symbol;Acc:HGNC:4708] | 2356 | 0.320 | 0.0061 | No |
| 29 | GH1 | growth hormone 1 [Source:HGNC Symbol;Acc:HGNC:4261] | 2456 | 0.308 | 0.0026 | No |
| 30 | CTSG | cathepsin G [Source:HGNC Symbol;Acc:HGNC:2532] | 2457 | 0.307 | 0.0069 | No |
| 31 | FPR3 | formyl peptide receptor 3 [Source:HGNC Symbol;Acc:HGNC:3828] | 2577 | 0.293 | 0.0017 | No |
| 32 | HCRTR1 | hypocretin receptor 1 [Source:HGNC Symbol;Acc:HGNC:4848] | 2644 | 0.286 | 0.0005 | No |
| 33 | GRIK2 | glutamate ionotropic receptor kainate type subunit 2 [Source:HGNC Symbol;Acc:HGNC:4580] | 2650 | 0.285 | 0.0041 | No |
| 34 | GHRHR | growth hormone releasing hormone receptor [Source:HGNC Symbol;Acc:HGNC:4266] | 2721 | 0.278 | 0.0025 | No |
| 35 | GABRP | gamma-aminobutyric acid type A receptor subunit pi [Source:HGNC Symbol;Acc:HGNC:4089] | 2847 | 0.266 | -0.0036 | No |
| 36 | P2RX5 | purinergic receptor P2X 5 [Source:HGNC Symbol;Acc:HGNC:8536] | 2937 | 0.257 | -0.0070 | No |
| 37 | CHRNA3 | cholinergic receptor nicotinic alpha 3 subunit [Source:HGNC Symbol;Acc:HGNC:1957] | 2995 | 0.250 | -0.0080 | No |
| 38 | CHRNA5 | cholinergic receptor nicotinic alpha 5 subunit [Source:HGNC Symbol;Acc:HGNC:1959] | 3128 | 0.237 | -0.0151 | No |
| 39 | OPRL1 | opioid related nociceptin receptor 1 [Source:HGNC Symbol;Acc:HGNC:8155] | 3166 | 0.234 | -0.0147 | No |
| 40 | GLRA2 | glycine receptor alpha 2 [Source:HGNC Symbol;Acc:HGNC:4327] | 3234 | 0.227 | -0.0168 | No |
| 41 | HTR1E | 5-hydroxytryptamine receptor 1E [Source:HGNC Symbol;Acc:HGNC:5291] | 3245 | 0.226 | -0.0144 | No |
| 42 | NPY1R | neuropeptide Y receptor Y1 [Source:HGNC Symbol;Acc:HGNC:7956] | 3249 | 0.226 | -0.0114 | No |
| 43 | DRD4 | dopamine receptor D4 [Source:HGNC Symbol;Acc:HGNC:3025] | 3267 | 0.224 | -0.0096 | No |
| 44 | GRM1 | glutamate metabotropic receptor 1 [Source:HGNC Symbol;Acc:HGNC:4593] | 3313 | 0.220 | -0.0100 | No |
| 45 | GABRG2 | gamma-aminobutyric acid type A receptor subunit gamma2 [Source:HGNC Symbol;Acc:HGNC:4087] | 3350 | 0.216 | -0.0098 | No |
| 46 | VIPR1 | vasoactive intestinal peptide receptor 1 [Source:HGNC Symbol;Acc:HGNC:12694] | 3394 | 0.212 | -0.0102 | No |
| 47 | CHRM2 | cholinergic receptor muscarinic 2 [Source:HGNC Symbol;Acc:HGNC:1951] | 3473 | 0.205 | -0.0135 | No |
| 48 | TACR3 | tachykinin receptor 3 [Source:HGNC Symbol;Acc:HGNC:11528] | 3527 | 0.200 | -0.0149 | No |
| 49 | MAS1 | "MAS1 proto-oncogene, G protein-coupled receptor [Source:HGNC Symbol;Acc:HGNC:6899]" | 3629 | 0.191 | -0.0202 | No |
| 50 | CSH1 | chorionic somatomammotropin hormone 1 [Source:HGNC Symbol;Acc:HGNC:2440] | 3634 | 0.191 | -0.0178 | No |
| 51 | OPRD1 | opioid receptor delta 1 [Source:HGNC Symbol;Acc:HGNC:8153] | 3663 | 0.189 | -0.0173 | No |
| 52 | CNR2 | cannabinoid receptor 2 [Source:HGNC Symbol;Acc:HGNC:2160] | 3669 | 0.188 | -0.0151 | No |
| 53 | TAAR5 | trace amine associated receptor 5 [Source:HGNC Symbol;Acc:HGNC:30236] | 3716 | 0.184 | -0.0161 | No |
| 54 | ADORA2A | adenosine A2a receptor [Source:HGNC Symbol;Acc:HGNC:263] | 3870 | 0.168 | -0.0258 | No |
| 55 | HTR2C | 5-hydroxytryptamine receptor 2C [Source:HGNC Symbol;Acc:HGNC:5295] | 3873 | 0.167 | -0.0236 | No |
| 56 | GLRA1 | glycine receptor alpha 1 [Source:HGNC Symbol;Acc:HGNC:4326] | 3890 | 0.165 | -0.0225 | No |
| 57 | CHRNB4 | cholinergic receptor nicotinic beta 4 subunit [Source:HGNC Symbol;Acc:HGNC:1964] | 3973 | 0.158 | -0.0268 | No |
| 58 | P2RX2 | purinergic receptor P2X 2 [Source:HGNC Symbol;Acc:HGNC:15459] | 4029 | 0.153 | -0.0290 | No |
| 59 | PTAFR | platelet activating factor receptor [Source:HGNC Symbol;Acc:HGNC:9582] | 4069 | 0.149 | -0.0300 | No |
| 60 | CHRNA1 | cholinergic receptor nicotinic alpha 1 subunit [Source:HGNC Symbol;Acc:HGNC:1955] | 4108 | 0.145 | -0.0309 | No |
| 61 | MTNR1B | melatonin receptor 1B [Source:HGNC Symbol;Acc:HGNC:7464] | 4133 | 0.143 | -0.0308 | No |
| 62 | PRLR | prolactin receptor [Source:HGNC Symbol;Acc:HGNC:9446] | 4323 | 0.126 | -0.0439 | No |
| 63 | P2RY6 | pyrimidinergic receptor P2Y6 [Source:HGNC Symbol;Acc:HGNC:8543] | 4374 | 0.123 | -0.0461 | No |
| 64 | P2RX7 | purinergic receptor P2X 7 [Source:HGNC Symbol;Acc:HGNC:8537] | 4415 | 0.120 | -0.0476 | No |
| 65 | CHRNA7 | cholinergic receptor nicotinic alpha 7 subunit [Source:HGNC Symbol;Acc:HGNC:1960] | 4475 | 0.115 | -0.0506 | No |
| 66 | FSHB | follicle stimulating hormone subunit beta [Source:HGNC Symbol;Acc:HGNC:3964] | 4502 | 0.113 | -0.0511 | No |
| 67 | ADRA1A | adrenoceptor alpha 1A [Source:HGNC Symbol;Acc:HGNC:277] | 4556 | 0.109 | -0.0538 | No |
| 68 | EDNRA | endothelin receptor type A [Source:HGNC Symbol;Acc:HGNC:3179] | 4663 | 0.099 | -0.0607 | No |
| 69 | F2 | "coagulation factor II, thrombin [Source:HGNC Symbol;Acc:HGNC:3535]" | 4745 | 0.092 | -0.0658 | No |
| 70 | GABBR2 | gamma-aminobutyric acid type B receptor subunit 2 [Source:HGNC Symbol;Acc:HGNC:4507] | 4872 | 0.082 | -0.0746 | No |
| 71 | LPAR3 | lysophosphatidic acid receptor 3 [Source:HGNC Symbol;Acc:HGNC:14298] | 4885 | 0.081 | -0.0745 | No |
| 72 | P2RY14 | purinergic receptor P2Y14 [Source:HGNC Symbol;Acc:HGNC:16442] | 4911 | 0.079 | -0.0753 | No |
| 73 | P2RY11 | purinergic receptor P2Y11 [Source:HGNC Symbol;Acc:HGNC:8540] | 4936 | 0.077 | -0.0761 | No |
| 74 | GRM5 | glutamate metabotropic receptor 5 [Source:HGNC Symbol;Acc:HGNC:4597] | 4941 | 0.077 | -0.0753 | No |
| 75 | GPR50 | G protein-coupled receptor 50 [Source:HGNC Symbol;Acc:HGNC:4506] | 4953 | 0.076 | -0.0751 | No |
| 76 | TSHR | thyroid stimulating hormone receptor [Source:HGNC Symbol;Acc:HGNC:12373] | 5112 | 0.063 | -0.0867 | No |
| 77 | ADRA1B | adrenoceptor alpha 1B [Source:HGNC Symbol;Acc:HGNC:278] | 5275 | 0.050 | -0.0988 | No |
| 78 | CHRM4 | cholinergic receptor muscarinic 4 [Source:HGNC Symbol;Acc:HGNC:1953] | 5312 | 0.046 | -0.1010 | No |
| 79 | TAAR2 | trace amine associated receptor 2 [Source:HGNC Symbol;Acc:HGNC:4514] | 5417 | 0.039 | -0.1087 | No |
| 80 | PRL | prolactin [Source:HGNC Symbol;Acc:HGNC:9445] | 5490 | 0.033 | -0.1139 | No |
| 81 | GABRB3 | gamma-aminobutyric acid type A receptor subunit beta3 [Source:HGNC Symbol;Acc:HGNC:4083] | 5606 | 0.023 | -0.1227 | No |
| 82 | GABRA5 | gamma-aminobutyric acid type A receptor subunit alpha5 [Source:HGNC Symbol;Acc:HGNC:4079] | 5629 | 0.022 | -0.1241 | No |
| 83 | THRA | thyroid hormone receptor alpha [Source:HGNC Symbol;Acc:HGNC:11796] | 5638 | 0.021 | -0.1244 | No |
| 84 | DRD5 | dopamine receptor D5 [Source:HGNC Symbol;Acc:HGNC:3026] | 5659 | 0.019 | -0.1258 | No |
| 85 | LHCGR | luteinizing hormone/choriogonadotropin receptor [Source:HGNC Symbol;Acc:HGNC:6585] | 5679 | 0.017 | -0.1270 | No |
| 86 | FSHR | follicle stimulating hormone receptor [Source:HGNC Symbol;Acc:HGNC:3969] | 5757 | 0.011 | -0.1329 | No |
| 87 | GABRA2 | gamma-aminobutyric acid type A receptor subunit alpha2 [Source:HGNC Symbol;Acc:HGNC:4076] | 5875 | 0.004 | -0.1421 | No |
| 88 | HTR1D | 5-hydroxytryptamine receptor 1D [Source:HGNC Symbol;Acc:HGNC:5289] | 5888 | 0.003 | -0.1431 | No |
| 89 | NPY4R | neuropeptide Y receptor Y4 [Source:HGNC Symbol;Acc:HGNC:9329] | 5894 | 0.002 | -0.1434 | No |
| 90 | GNRHR | gonadotropin releasing hormone receptor [Source:HGNC Symbol;Acc:HGNC:4421] | 5903 | 0.002 | -0.1440 | No |
| 91 | MC3R | melanocortin 3 receptor [Source:HGNC Symbol;Acc:HGNC:6931] | 5910 | 0.002 | -0.1445 | No |
| 92 | MC4R | melanocortin 4 receptor [Source:HGNC Symbol;Acc:HGNC:6932] | 5918 | 0.001 | -0.1450 | No |
| 93 | GABRR1 | gamma-aminobutyric acid type A receptor subunit rho1 [Source:HGNC Symbol;Acc:HGNC:4090] | 5920 | 0.001 | -0.1451 | No |
| 94 | MC1R | melanocortin 1 receptor [Source:HGNC Symbol;Acc:HGNC:6929] | 5923 | 0.001 | -0.1452 | No |
| 95 | MC5R | melanocortin 5 receptor [Source:HGNC Symbol;Acc:HGNC:6933] | 5933 | 0.000 | -0.1459 | No |
| 96 | TRPV1 | transient receptor potential cation channel subfamily V member 1 [Source:HGNC Symbol;Acc:HGNC:12716] | 6032 | -0.007 | -0.1536 | No |
| 97 | NPY5R | neuropeptide Y receptor Y5 [Source:HGNC Symbol;Acc:HGNC:7958] | 6064 | -0.009 | -0.1559 | No |
| 98 | AGTR1 | angiotensin II receptor type 1 [Source:HGNC Symbol;Acc:HGNC:336] | 6122 | -0.014 | -0.1602 | No |
| 99 | GABRB2 | gamma-aminobutyric acid type A receptor subunit beta2 [Source:HGNC Symbol;Acc:HGNC:4082] | 6129 | -0.015 | -0.1605 | No |
| 100 | MCHR1 | melanin concentrating hormone receptor 1 [Source:HGNC Symbol;Acc:HGNC:4479] | 6242 | -0.024 | -0.1690 | No |
| 101 | BDKRB2 | bradykinin receptor B2 [Source:HGNC Symbol;Acc:HGNC:1030] | 6276 | -0.026 | -0.1712 | No |
| 102 | OPRM1 | opioid receptor mu 1 [Source:HGNC Symbol;Acc:HGNC:8156] | 6351 | -0.032 | -0.1766 | No |
| 103 | CRHR1 | corticotropin releasing hormone receptor 1 [Source:HGNC Symbol;Acc:HGNC:2357] | 6390 | -0.034 | -0.1792 | No |
| 104 | GRIA3 | glutamate ionotropic receptor AMPA type subunit 3 [Source:HGNC Symbol;Acc:HGNC:4573] | 6454 | -0.039 | -0.1836 | No |
| 105 | ADRA2B | adrenoceptor alpha 2B [Source:HGNC Symbol;Acc:HGNC:282] | 6513 | -0.043 | -0.1876 | No |
| 106 | PTGER1 | prostaglandin E receptor 1 [Source:HGNC Symbol;Acc:HGNC:9593] | 6527 | -0.044 | -0.1880 | No |
| 107 | NTSR2 | neurotensin receptor 2 [Source:HGNC Symbol;Acc:HGNC:8040] | 6557 | -0.046 | -0.1896 | No |
| 108 | PRSS1 | serine protease 1 [Source:HGNC Symbol;Acc:HGNC:9475] | 6571 | -0.047 | -0.1900 | No |
| 109 | TBXA2R | thromboxane A2 receptor [Source:HGNC Symbol;Acc:HGNC:11608] | 6651 | -0.053 | -0.1955 | No |
| 110 | CRHR2 | corticotropin releasing hormone receptor 2 [Source:HGNC Symbol;Acc:HGNC:2358] | 6672 | -0.054 | -0.1963 | No |
| 111 | GRM7 | glutamate metabotropic receptor 7 [Source:HGNC Symbol;Acc:HGNC:4599] | 6694 | -0.055 | -0.1972 | No |
| 112 | CHRNB3 | cholinergic receptor nicotinic beta 3 subunit [Source:HGNC Symbol;Acc:HGNC:1963] | 6703 | -0.056 | -0.1970 | No |
| 113 | GHR | growth hormone receptor [Source:HGNC Symbol;Acc:HGNC:4263] | 6740 | -0.058 | -0.1990 | No |
| 114 | GRIK3 | glutamate ionotropic receptor kainate type subunit 3 [Source:HGNC Symbol;Acc:HGNC:4581] | 6744 | -0.059 | -0.1984 | No |
| 115 | CYSLTR2 | cysteinyl leukotriene receptor 2 [Source:HGNC Symbol;Acc:HGNC:18274] | 6965 | -0.077 | -0.2147 | No |
| 116 | GIPR | gastric inhibitory polypeptide receptor [Source:HGNC Symbol;Acc:HGNC:4271] | 6979 | -0.078 | -0.2147 | No |
| 117 | GH2 | growth hormone 2 [Source:HGNC Symbol;Acc:HGNC:4262] | 6993 | -0.079 | -0.2146 | No |
| 118 | P2RY10 | P2Y receptor family member 10 [Source:HGNC Symbol;Acc:HGNC:19906] | 7014 | -0.081 | -0.2150 | No |
| 119 | PTH1R | parathyroid hormone 1 receptor [Source:HGNC Symbol;Acc:HGNC:9608] | 7095 | -0.087 | -0.2201 | No |
| 120 | DRD3 | dopamine receptor D3 [Source:HGNC Symbol;Acc:HGNC:3024] | 7101 | -0.088 | -0.2193 | No |
| 121 | ADORA1 | adenosine A1 receptor [Source:HGNC Symbol;Acc:HGNC:262] | 7165 | -0.093 | -0.2229 | No |
| 122 | MC2R | melanocortin 2 receptor [Source:HGNC Symbol;Acc:HGNC:6930] | 7169 | -0.093 | -0.2218 | No |
| 123 | GABRA1 | gamma-aminobutyric acid type A receptor subunit alpha1 [Source:HGNC Symbol;Acc:HGNC:4075] | 7239 | -0.098 | -0.2259 | No |
| 124 | P2RY13 | purinergic receptor P2Y13 [Source:HGNC Symbol;Acc:HGNC:4537] | 7242 | -0.099 | -0.2247 | No |
| 125 | TSHB | thyroid stimulating hormone subunit beta [Source:HGNC Symbol;Acc:HGNC:12372] | 7342 | -0.105 | -0.2310 | No |
| 126 | GRPR | gastrin releasing peptide receptor [Source:HGNC Symbol;Acc:HGNC:4609] | 7361 | -0.107 | -0.2309 | No |
| 127 | OXTR | oxytocin receptor [Source:HGNC Symbol;Acc:HGNC:8529] | 7534 | -0.121 | -0.2428 | No |
| 128 | GRM4 | glutamate metabotropic receptor 4 [Source:HGNC Symbol;Acc:HGNC:4596] | 7658 | -0.131 | -0.2507 | No |
| 129 | GCGR | glucagon receptor [Source:HGNC Symbol;Acc:HGNC:4192] | 7697 | -0.134 | -0.2518 | No |
| 130 | LPAR1 | lysophosphatidic acid receptor 1 [Source:HGNC Symbol;Acc:HGNC:3166] | 7807 | -0.142 | -0.2584 | No |
| 131 | MTNR1A | melatonin receptor 1A [Source:HGNC Symbol;Acc:HGNC:7463] | 7817 | -0.142 | -0.2571 | No |
| 132 | GPR35 | G protein-coupled receptor 35 [Source:HGNC Symbol;Acc:HGNC:4492] | 7831 | -0.143 | -0.2561 | No |
| 133 | GRIK5 | glutamate ionotropic receptor kainate type subunit 5 [Source:HGNC Symbol;Acc:HGNC:4583] | 7912 | -0.148 | -0.2603 | No |
| 134 | APLNR | apelin receptor [Source:HGNC Symbol;Acc:HGNC:339] | 8001 | -0.155 | -0.2651 | No |
| 135 | CHRM3 | cholinergic receptor muscarinic 3 [Source:HGNC Symbol;Acc:HGNC:1952] | 8034 | -0.158 | -0.2654 | No |
| 136 | CHRNA2 | cholinergic receptor nicotinic alpha 2 subunit [Source:HGNC Symbol;Acc:HGNC:1956] | 8035 | -0.158 | -0.2632 | No |
| 137 | DRD2 | dopamine receptor D2 [Source:HGNC Symbol;Acc:HGNC:3023] | 8086 | -0.162 | -0.2648 | No |
| 138 | BRS3 | bombesin receptor subtype 3 [Source:HGNC Symbol;Acc:HGNC:1113] | 8109 | -0.164 | -0.2642 | No |
| 139 | HRH1 | histamine receptor H1 [Source:HGNC Symbol;Acc:HGNC:5182] | 8146 | -0.166 | -0.2647 | No |
| 140 | S1PR2 | sphingosine-1-phosphate receptor 2 [Source:HGNC Symbol;Acc:HGNC:3169] | 8154 | -0.167 | -0.2629 | No |
| 141 | TACR2 | tachykinin receptor 2 [Source:HGNC Symbol;Acc:HGNC:11527] | 8160 | -0.168 | -0.2610 | No |
| 142 | ADRB1 | adrenoceptor beta 1 [Source:HGNC Symbol;Acc:HGNC:285] | 8212 | -0.172 | -0.2626 | No |
| 143 | NPY2R | neuropeptide Y receptor Y2 [Source:HGNC Symbol;Acc:HGNC:7957] | 8295 | -0.178 | -0.2665 | No |
| 144 | GRIK4 | glutamate ionotropic receptor kainate type subunit 4 [Source:HGNC Symbol;Acc:HGNC:4582] | 8296 | -0.178 | -0.2640 | No |
| 145 | GRIA4 | glutamate ionotropic receptor AMPA type subunit 4 [Source:HGNC Symbol;Acc:HGNC:4574] | 8390 | -0.185 | -0.2687 | No |
| 146 | GRM2 | glutamate metabotropic receptor 2 [Source:HGNC Symbol;Acc:HGNC:4594] | 8425 | -0.187 | -0.2688 | No |
| 147 | F2R | coagulation factor II thrombin receptor [Source:HGNC Symbol;Acc:HGNC:3537] | 8447 | -0.190 | -0.2678 | No |
| 148 | GHSR | growth hormone secretagogue receptor [Source:HGNC Symbol;Acc:HGNC:4267] | 8472 | -0.191 | -0.2670 | No |
| 149 | LEPR | leptin receptor [Source:HGNC Symbol;Acc:HGNC:6554] | 8479 | -0.192 | -0.2647 | No |
| 150 | HCRTR2 | hypocretin receptor 2 [Source:HGNC Symbol;Acc:HGNC:4849] | 8495 | -0.193 | -0.2632 | No |
| 151 | CGA | "glycoprotein hormones, alpha polypeptide [Source:HGNC Symbol;Acc:HGNC:1885]" | 8583 | -0.200 | -0.2672 | No |
| 152 | GLP2R | glucagon like peptide 2 receptor [Source:HGNC Symbol;Acc:HGNC:4325] | 8606 | -0.202 | -0.2661 | No |
| 153 | LPAR4 | lysophosphatidic acid receptor 4 [Source:HGNC Symbol;Acc:HGNC:4478] | 8617 | -0.202 | -0.2640 | No |
| 154 | F2RL1 | F2R like trypsin receptor 1 [Source:HGNC Symbol;Acc:HGNC:3538] | 8631 | -0.203 | -0.2622 | No |
| 155 | CHRNA10 | cholinergic receptor nicotinic alpha 10 subunit [Source:HGNC Symbol;Acc:HGNC:13800] | 8643 | -0.204 | -0.2602 | No |
| 156 | CHRND | cholinergic receptor nicotinic delta subunit [Source:HGNC Symbol;Acc:HGNC:1965] | 8651 | -0.205 | -0.2579 | No |
| 157 | LEP | leptin [Source:HGNC Symbol;Acc:HGNC:6553] | 8740 | -0.212 | -0.2618 | No |
| 158 | HRH4 | histamine receptor H4 [Source:HGNC Symbol;Acc:HGNC:17383] | 8852 | -0.221 | -0.2675 | No |
| 159 | PRSS2 | serine protease 2 [Source:HGNC Symbol;Acc:HGNC:9483] | 8969 | -0.230 | -0.2734 | Yes |
| 160 | GRID2 | glutamate ionotropic receptor delta type subunit 2 [Source:HGNC Symbol;Acc:HGNC:4576] | 8974 | -0.230 | -0.2705 | Yes |
| 161 | AGTR2 | angiotensin II receptor type 2 [Source:HGNC Symbol;Acc:HGNC:338] | 9009 | -0.232 | -0.2699 | Yes |
| 162 | OPRK1 | opioid receptor kappa 1 [Source:HGNC Symbol;Acc:HGNC:8154] | 9046 | -0.235 | -0.2694 | Yes |
| 163 | NPFFR1 | neuropeptide FF receptor 1 [Source:HGNC Symbol;Acc:HGNC:17425] | 9058 | -0.236 | -0.2669 | Yes |
| 164 | TACR1 | tachykinin receptor 1 [Source:HGNC Symbol;Acc:HGNC:11526] | 9090 | -0.239 | -0.2660 | Yes |
| 165 | CHRNG | cholinergic receptor nicotinic gamma subunit [Source:HGNC Symbol;Acc:HGNC:1967] | 9101 | -0.239 | -0.2634 | Yes |
| 166 | FPR1 | formyl peptide receptor 1 [Source:HGNC Symbol;Acc:HGNC:3826] | 9110 | -0.240 | -0.2606 | Yes |
| 167 | GABRG3 | gamma-aminobutyric acid type A receptor subunit gamma3 [Source:HGNC Symbol;Acc:HGNC:4088] | 9164 | -0.245 | -0.2614 | Yes |
| 168 | PTH2R | parathyroid hormone 2 receptor [Source:HGNC Symbol;Acc:HGNC:9609] | 9197 | -0.247 | -0.2604 | Yes |
| 169 | ADORA2B | adenosine A2b receptor [Source:HGNC Symbol;Acc:HGNC:264] | 9255 | -0.252 | -0.2613 | Yes |
| 170 | SSTR5 | somatostatin receptor 5 [Source:HGNC Symbol;Acc:HGNC:11334] | 9272 | -0.253 | -0.2590 | Yes |
| 171 | P2RY2 | purinergic receptor P2Y2 [Source:HGNC Symbol;Acc:HGNC:8541] | 9470 | -0.271 | -0.2708 | Yes |
| 172 | GRIN2C | glutamate ionotropic receptor NMDA type subunit 2C [Source:HGNC Symbol;Acc:HGNC:4587] | 9515 | -0.275 | -0.2704 | Yes |
| 173 | CALCRL | calcitonin receptor like receptor [Source:HGNC Symbol;Acc:HGNC:16709] | 9584 | -0.282 | -0.2718 | Yes |
| 174 | S1PR4 | sphingosine-1-phosphate receptor 4 [Source:HGNC Symbol;Acc:HGNC:3170] | 9596 | -0.283 | -0.2686 | Yes |
| 175 | NPBWR2 | neuropeptides B and W receptor 2 [Source:HGNC Symbol;Acc:HGNC:4530] | 9622 | -0.285 | -0.2666 | Yes |
| 176 | GALR3 | galanin receptor 3 [Source:HGNC Symbol;Acc:HGNC:4134] | 9722 | -0.294 | -0.2702 | Yes |
| 177 | ADRA2C | adrenoceptor alpha 2C [Source:HGNC Symbol;Acc:HGNC:283] | 9791 | -0.301 | -0.2714 | Yes |
| 178 | SSTR1 | somatostatin receptor 1 [Source:HGNC Symbol;Acc:HGNC:11330] | 9803 | -0.302 | -0.2680 | Yes |
| 179 | THRB | thyroid hormone receptor beta [Source:HGNC Symbol;Acc:HGNC:11799] | 9820 | -0.303 | -0.2650 | Yes |
| 180 | GRM6 | glutamate metabotropic receptor 6 [Source:HGNC Symbol;Acc:HGNC:4598] | 9827 | -0.304 | -0.2612 | Yes |
| 181 | FPR2 | formyl peptide receptor 2 [Source:HGNC Symbol;Acc:HGNC:3827] | 9857 | -0.307 | -0.2591 | Yes |
| 182 | P2RY1 | purinergic receptor P2Y1 [Source:HGNC Symbol;Acc:HGNC:8539] | 9925 | -0.314 | -0.2600 | Yes |
| 183 | P2RX1 | purinergic receptor P2X 1 [Source:HGNC Symbol;Acc:HGNC:8533] | 9926 | -0.314 | -0.2555 | Yes |
| 184 | CALCR | calcitonin receptor [Source:HGNC Symbol;Acc:HGNC:1440] | 9937 | -0.315 | -0.2519 | Yes |
| 185 | P2RY4 | pyrimidinergic receptor P2Y4 [Source:HGNC Symbol;Acc:HGNC:8542] | 9978 | -0.319 | -0.2505 | Yes |
| 186 | HTR1A | 5-hydroxytryptamine receptor 1A [Source:HGNC Symbol;Acc:HGNC:5286] | 10008 | -0.322 | -0.2483 | Yes |
| 187 | ADRA1D | adrenoceptor alpha 1D [Source:HGNC Symbol;Acc:HGNC:280] | 10031 | -0.325 | -0.2454 | Yes |
| 188 | PTGER3 | prostaglandin E receptor 3 [Source:HGNC Symbol;Acc:HGNC:9595] | 10073 | -0.328 | -0.2440 | Yes |
| 189 | GABRD | gamma-aminobutyric acid type A receptor subunit delta [Source:HGNC Symbol;Acc:HGNC:4084] | 10140 | -0.335 | -0.2445 | Yes |
| 190 | CHRNA9 | cholinergic receptor nicotinic alpha 9 subunit [Source:HGNC Symbol;Acc:HGNC:14079] | 10213 | -0.342 | -0.2454 | Yes |
| 191 | CHRNA6 | cholinergic receptor nicotinic alpha 6 subunit [Source:HGNC Symbol;Acc:HGNC:15963] | 10240 | -0.345 | -0.2426 | Yes |
| 192 | GRIK1 | glutamate ionotropic receptor kainate type subunit 1 [Source:HGNC Symbol;Acc:HGNC:4579] | 10274 | -0.348 | -0.2402 | Yes |
| 193 | VIPR2 | vasoactive intestinal peptide receptor 2 [Source:HGNC Symbol;Acc:HGNC:12695] | 10358 | -0.357 | -0.2418 | Yes |
| 194 | HTR7 | 5-hydroxytryptamine receptor 7 [Source:HGNC Symbol;Acc:HGNC:5302] | 10399 | -0.360 | -0.2398 | Yes |
| 195 | MLNR | motilin receptor [Source:HGNC Symbol;Acc:HGNC:4495] | 10434 | -0.365 | -0.2374 | Yes |
| 196 | NMBR | neuromedin B receptor [Source:HGNC Symbol;Acc:HGNC:7843] | 10503 | -0.373 | -0.2375 | Yes |
| 197 | F2RL2 | coagulation factor II thrombin receptor like 2 [Source:HGNC Symbol;Acc:HGNC:3539] | 10524 | -0.376 | -0.2337 | Yes |
| 198 | GABRR2 | gamma-aminobutyric acid type A receptor subunit rho2 [Source:HGNC Symbol;Acc:HGNC:4091] | 10543 | -0.377 | -0.2298 | Yes |
| 199 | PTGER2 | prostaglandin E receptor 2 [Source:HGNC Symbol;Acc:HGNC:9594] | 10596 | -0.383 | -0.2285 | Yes |
| 200 | CHRNE | cholinergic receptor nicotinic epsilon subunit [Source:HGNC Symbol;Acc:HGNC:1966] | 10620 | -0.385 | -0.2249 | Yes |
| 201 | GABRE | gamma-aminobutyric acid type A receptor subunit epsilon [Source:HGNC Symbol;Acc:HGNC:4085] | 10672 | -0.390 | -0.2234 | Yes |
| 202 | CHRM5 | cholinergic receptor muscarinic 5 [Source:HGNC Symbol;Acc:HGNC:1954] | 10725 | -0.396 | -0.2219 | Yes |
| 203 | SCTR | secretin receptor [Source:HGNC Symbol;Acc:HGNC:10608] | 10726 | -0.396 | -0.2163 | Yes |
| 204 | S1PR5 | sphingosine-1-phosphate receptor 5 [Source:HGNC Symbol;Acc:HGNC:14299] | 10785 | -0.404 | -0.2152 | Yes |
| 205 | ADRA2A | adrenoceptor alpha 2A [Source:HGNC Symbol;Acc:HGNC:281] | 10790 | -0.404 | -0.2098 | Yes |
| 206 | CCKBR | cholecystokinin B receptor [Source:HGNC Symbol;Acc:HGNC:1571] | 10842 | -0.410 | -0.2081 | Yes |
| 207 | PLG | plasminogen [Source:HGNC Symbol;Acc:HGNC:9071] | 10939 | -0.423 | -0.2097 | Yes |
| 208 | BDKRB1 | bradykinin receptor B1 [Source:HGNC Symbol;Acc:HGNC:1029] | 10951 | -0.424 | -0.2045 | Yes |
| 209 | HTR1B | 5-hydroxytryptamine receptor 1B [Source:HGNC Symbol;Acc:HGNC:5287] | 10971 | -0.427 | -0.2000 | Yes |
| 210 | C5AR1 | complement C5a receptor 1 [Source:HGNC Symbol;Acc:HGNC:1338] | 10978 | -0.427 | -0.1944 | Yes |
| 211 | GLRA3 | glycine receptor alpha 3 [Source:HGNC Symbol;Acc:HGNC:4328] | 10999 | -0.429 | -0.1900 | Yes |
| 212 | CHRNB2 | cholinergic receptor nicotinic beta 2 subunit [Source:HGNC Symbol;Acc:HGNC:1962] | 11028 | -0.434 | -0.1860 | Yes |
| 213 | HTR6 | 5-hydroxytryptamine receptor 6 [Source:HGNC Symbol;Acc:HGNC:5301] | 11034 | -0.435 | -0.1803 | Yes |
| 214 | CNR1 | cannabinoid receptor 1 [Source:HGNC Symbol;Acc:HGNC:2159] | 11181 | -0.454 | -0.1854 | Yes |
| 215 | S1PR1 | sphingosine-1-phosphate receptor 1 [Source:HGNC Symbol;Acc:HGNC:3165] | 11230 | -0.461 | -0.1827 | Yes |
| 216 | HTR1F | 5-hydroxytryptamine receptor 1F [Source:HGNC Symbol;Acc:HGNC:5292] | 11290 | -0.470 | -0.1807 | Yes |
| 217 | GRIN2D | glutamate ionotropic receptor NMDA type subunit 2D [Source:HGNC Symbol;Acc:HGNC:4588] | 11306 | -0.472 | -0.1752 | Yes |
| 218 | LPAR2 | lysophosphatidic acid receptor 2 [Source:HGNC Symbol;Acc:HGNC:3168] | 11338 | -0.476 | -0.1710 | Yes |
| 219 | GRM3 | glutamate metabotropic receptor 3 [Source:HGNC Symbol;Acc:HGNC:4595] | 11351 | -0.478 | -0.1652 | Yes |
| 220 | GRIN2B | glutamate ionotropic receptor NMDA type subunit 2B [Source:HGNC Symbol;Acc:HGNC:4586] | 11487 | -0.500 | -0.1688 | Yes |
| 221 | AVPR2 | arginine vasopressin receptor 2 [Source:HGNC Symbol;Acc:HGNC:897] | 11512 | -0.502 | -0.1636 | Yes |
| 222 | PRSS3 | serine protease 3 [Source:HGNC Symbol;Acc:HGNC:9486] | 11563 | -0.509 | -0.1603 | Yes |
| 223 | CYSLTR1 | cysteinyl leukotriene receptor 1 [Source:HGNC Symbol;Acc:HGNC:17451] | 11625 | -0.521 | -0.1578 | Yes |
| 224 | GABBR1 | gamma-aminobutyric acid type B receptor subunit 1 [Source:HGNC Symbol;Acc:HGNC:4070] | 11641 | -0.524 | -0.1516 | Yes |
| 225 | GRM8 | glutamate metabotropic receptor 8 [Source:HGNC Symbol;Acc:HGNC:4600] | 11654 | -0.525 | -0.1451 | Yes |
| 226 | ADRB3 | adrenoceptor beta 3 [Source:HGNC Symbol;Acc:HGNC:288] | 11691 | -0.531 | -0.1404 | Yes |
| 227 | HRH3 | histamine receptor H3 [Source:HGNC Symbol;Acc:HGNC:5184] | 11827 | -0.554 | -0.1433 | Yes |
| 228 | GALR1 | galanin receptor 1 [Source:HGNC Symbol;Acc:HGNC:4132] | 11851 | -0.560 | -0.1372 | Yes |
| 229 | HRH2 | histamine receptor H2 [Source:HGNC Symbol;Acc:HGNC:5183] | 11879 | -0.567 | -0.1313 | Yes |
| 230 | P2RX3 | purinergic receptor P2X 3 [Source:HGNC Symbol;Acc:HGNC:8534] | 11903 | -0.572 | -0.1250 | Yes |
| 231 | GALR2 | galanin receptor 2 [Source:HGNC Symbol;Acc:HGNC:4133] | 12072 | -0.612 | -0.1297 | Yes |
| 232 | EDNRB | endothelin receptor type B [Source:HGNC Symbol;Acc:HGNC:3180] | 12075 | -0.613 | -0.1212 | Yes |
| 233 | GRIA1 | glutamate ionotropic receptor AMPA type subunit 1 [Source:HGNC Symbol;Acc:HGNC:4571] | 12149 | -0.631 | -0.1180 | Yes |
| 234 | SSTR2 | somatostatin receptor 2 [Source:HGNC Symbol;Acc:HGNC:11331] | 12181 | -0.639 | -0.1114 | Yes |
| 235 | TRHR | thyrotropin releasing hormone receptor [Source:HGNC Symbol;Acc:HGNC:12299] | 12250 | -0.660 | -0.1075 | Yes |
| 236 | HTR4 | 5-hydroxytryptamine receptor 4 [Source:HGNC Symbol;Acc:HGNC:5299] | 12308 | -0.680 | -0.1024 | Yes |
| 237 | LTB4R | leukotriene B4 receptor [Source:HGNC Symbol;Acc:HGNC:6713] | 12472 | -0.737 | -0.1049 | Yes |
| 238 | PARD3 | par-3 family cell polarity regulator [Source:HGNC Symbol;Acc:HGNC:16051] | 12489 | -0.743 | -0.0956 | Yes |
| 239 | P2RX6 | purinergic receptor P2X 6 [Source:HGNC Symbol;Acc:HGNC:8538] | 12529 | -0.762 | -0.0879 | Yes |
| 240 | GLP1R | glucagon like peptide 1 receptor [Source:HGNC Symbol;Acc:HGNC:4324] | 12596 | -0.795 | -0.0819 | Yes |
| 241 | HTR5A | 5-hydroxytryptamine receptor 5A [Source:HGNC Symbol;Acc:HGNC:5300] | 12599 | -0.797 | -0.0708 | Yes |
| 242 | GRIN1 | glutamate ionotropic receptor NMDA type subunit 1 [Source:HGNC Symbol;Acc:HGNC:4584] | 12603 | -0.799 | -0.0598 | Yes |
| 243 | NTSR1 | neurotensin receptor 1 [Source:HGNC Symbol;Acc:HGNC:8039] | 12677 | -0.849 | -0.0535 | Yes |
| 244 | AVPR1A | arginine vasopressin receptor 1A [Source:HGNC Symbol;Acc:HGNC:895] | 12751 | -0.913 | -0.0464 | Yes |
| 245 | SSTR3 | somatostatin receptor 3 [Source:HGNC Symbol;Acc:HGNC:11332] | 12779 | -0.936 | -0.0353 | Yes |
| 246 | ADCYAP1R1 | ADCYAP receptor type I [Source:HGNC Symbol;Acc:HGNC:242] | 12790 | -0.952 | -0.0227 | Yes |
| 247 | LHB | luteinizing hormone subunit beta [Source:HGNC Symbol;Acc:HGNC:6584] | 12805 | -0.975 | -0.0100 | Yes |
| 248 | NMUR1 | neuromedin U receptor 1 [Source:HGNC Symbol;Acc:HGNC:4518] | 12887 | -1.233 | 0.0010 | Yes |
Table: GSEA details [plain text format]

  

Fig 2: KEGG\_NEUROACTIVE\_LIGAND\_RECEPTOR\_INTERACTION      
 Blue-Pink O' Gram in the Space of the Analyzed GeneSet

  

Fig 3: KEGG\_NEUROACTIVE\_LIGAND\_RECEPTOR\_INTERACTION: Random ES distribution      
 Gene set null distribution of ES for **KEGG\_NEUROACTIVE\_LIGAND\_RECEPTOR\_INTERACTION**
